# Clinical Effectiveness and Tolerability of Parenteral Iron Sucrose in the Second- and Third-Trimester Anemia: A Retrospective Record-Based Analysis

**DOI:** 10.7759/cureus.100587

**Published:** 2026-01-01

**Authors:** Vipul N Sarvaiya, Sajidali S Saiyad, Pooja Parmar, Roma Umakant Dubey, Tazean Zahoor Malik, Shreya Kattela, Alkeshkumar R Vara, Chetna Brijen Vadodariya

**Affiliations:** 1 Obstetrics and Gynaecology, Matushri Prabhaben Khodabhai Boghara Medical College and K.D. Parvadiya Multi-Speciality Hospital, Atkot, IND; 2 Physiology, Pacific Medical College and Hospital, Pacific Medical University, Udaipur, IND; 3 Obstetrics and Gynaecology, J and D Institute of Nursing, Surat, IND; 4 Obstetrics and Gynaecology, Aryavart Hospital, Meerut, IND; 5 Community Medicine, Government Medical College, Srinagar, Srinagar, IND; 6 Internal Medicine, Bhaskar Medical College, Hyderabad, IND; 7 Physiology, Nootan Medical College and Research Centre, Visnagar, IND; 8 Obstetrics and Gynaecology, Government Medical College, Maharaja Krishnakumarsinhji Bhavnagar University, Bhavnagar, IND

**Keywords:** antenatal anemia correction, hemoglobin response to iv iron, intravenous iron sucrose therapy, iron-deficiency anemia in pregnancy, late-gestation anemia management, maternal-fetal outcomes, microcytic hypochromic anemia, neonatal birth outcomes and anemia, parenteral iron supplementation, retrospective clinical effectiveness study

## Abstract

Background

Iron-deficiency anemia (IDA) is a leading cause of adverse maternal and neonatal outcomes, particularly in resource-limited settings where nutritional deficits and late antenatal presentation are common. When diagnosed in late gestation, oral iron therapy is often insufficient for timely hematologic correction.

Methods

This retrospective observational study reviewed medical records of 75 pregnant women between 26 and 32 weeks of gestation with hemoglobin <9 g/dL who had received intravenous iron sucrose as part of routine clinical care. Baseline and six-week post-treatment hematologic parameters, along with maternal delivery outcomes and neonatal indicators, were extracted from existing hospital records. Statistical analyses included paired t-tests, chi-square tests, and effect size estimation.

Results

Intravenous iron sucrose produced a substantial and statistically significant improvement in hematologic parameters, with clear correction of microcytic indices by the six-week follow-up. The therapy was well tolerated, with only minor infusion-related events and no serious adverse reactions. Maternal delivery patterns were largely favorable, and neonatal outcomes - including birthweight distribution and need for intensive care - were consistent with improved perinatal stability following effective anemia correction. The observed hematologic improvements were consistent across recorded demographic profiles, reinforcing the reliability of the findings in nutritionally vulnerable populations.

Conclusion

Intravenous iron sucrose provides rapid, clinically meaningful correction of IDA in late pregnancy. No serious adverse events were documented in the available medical records. These findings support its integration into antenatal anemia management, particularly where oral iron is inadequate or poorly tolerated, and highlight its potential to reduce anemia-associated maternal-fetal morbidity. The retrospective design further reflects the real-world clinical effectiveness of iron sucrose in late-gestation anemia.

## Introduction

Iron-deficiency anemia (IDA) remains the most prevalent hematological disorder complicating pregnancy and is a major contributor to maternal and perinatal morbidity worldwide, particularly in low- and middle-income countries [1]. Pregnancy is characterized by substantial hematologic adaptations - including expanded plasma volume, accelerated erythropoiesis, and increased fetal-placental iron transfer - that collectively heighten maternal iron requirements. When these demands are unmet, physiological hemodilution progresses to pathological anemia, defined by the World Health Organization as hemoglobin levels below 11 g/dL [2]. India continues to report some of the highest global burdens of IDA in pregnancy, driven by nutritional deficits, frequent pregnancies, low socioeconomic conditions, and a high prevalence of infections and parasitic diseases [3].

Effective management of IDA during pregnancy requires timely screening, nutritional counseling, and appropriate iron supplementation. While oral iron remains the conventional first-line therapy due to its accessibility, its effectiveness is often limited by gastrointestinal intolerance, poor adherence, and variable absorption - particularly problematic in moderate to severe anemia or when diagnosed late in gestation [4]. In such circumstances, rapid hematologic correction is essential to minimize obstetric risks and optimize fetal oxygenation, prompting consideration of parenteral formulations. Intravenous iron therapies have gained prominence for their predictable absorption and capacity to replenish iron stores swiftly, addressing limitations inherent to oral regimens [5].

Among available intravenous formulations, iron sucrose complex (ISC) has emerged as a widely adopted option due to its favorable safety profile, absence of dextran components, and low risk of anaphylaxis. ISC demonstrates reliable uptake through the reticuloendothelial system and produces consistent improvements in hemoglobin, hematocrit, and red-cell indices without the need for test dosing [6,7]. Multiple studies have reported faster hematologic responses and reduced transfusion requirements with ISC compared with oral iron, particularly in late-trimester anemia, where treatment windows are narrow [8-10]. Accordingly, intravenous iron sucrose is frequently used when rapid correction is required, and the treatment window is limited in late gestation.

The primary objective of this retrospective, record-based study was to evaluate the hematologic response to intravenous iron sucrose therapy in pregnant women between 26 and 32 weeks of gestation with hemoglobin levels <9 g/dL. Secondary objectives were to assess the documented tolerability profile and associated maternal delivery and neonatal outcomes. By utilizing existing hospital records spanning a nutritionally vulnerable population, this retrospective analysis provides real-world evidence on treatment effectiveness, delivery patterns, and neonatal outcomes. These findings aim to inform clinical practice in resource-limited settings and strengthen the role of parenteral iron sucrose in contemporary antenatal anemia management.

## Materials and methods

Study design

This investigation was conducted as a retrospective, observational study based on a review of existing hospital records. All pregnant women who had received intravenous iron sucrose for documented IDA were identified from antenatal case files, laboratory archives, and infusion registers. No prospective enrolment, randomization, or intervention was performed, and all treatments had already been administered as part of routine clinical care. The study assessed hematologic response, maternal delivery outcomes, and neonatal parameters using pre-existing data. 

Study setting

This was a single-center, retrospective, record-based, observational study conducted at a tertiary-care hospital serving predominantly low- and middle-income populations from surrounding rural and semi-urban regions. Medical records dated September 2009 to November 2011 were retrieved from the institutional medical record department and obstetric archives. All hematologic investigations, treatment details, and delivery/neonatal outcomes had been documented as part of routine antenatal care.

Study participants

Records of pregnant women between 26 and 32 weeks of gestation with hemoglobin levels <9 g/dL, who had received intravenous iron sucrose, were identified from institutional medical archives. Gestational age was determined from antenatal records using the last menstrual period, corroborated with ultrasound dating, as documented by the treating clinician. Inclusion required availability of both baseline and six-week post-treatment hematologic values, along with documented maternal delivery and neonatal outcomes. Records with incomplete laboratory data, uncertain anemia diagnosis, or missing treatment documentation were excluded. A total of 122 antenatal case files were screened using the same predefined data abstraction protocol. After application of the eligibility criteria, 75 complete records were included in the final analysis. IDA was identified based on hemoglobin concentration <9 g/dL in the presence of microcytic, hypochromic red-cell indices, particularly reduced mean corpuscular volume (MCV). Iron studies, including serum ferritin and serum iron, were not routinely available in the archived records due to cost constraints and, therefore, were not used for diagnostic confirmation. Only singleton pregnancies were included in the analysis. Major maternal comorbidities were not systematically evaluated due to the retrospective nature of the record review.

Sample size determination

As this was a retrospective, record-based study, no prospective sample size calculation was performed. All eligible antenatal case records with complete baseline, treatment, and follow-up data available during the study period were included in the analysis. A total of 75 eligible and complete case records were available in the hospital archives and were included in the final analysis. As this was a retrospective, record-based analysis, the sample size calculation represents a post hoc statistical justification rather than prospective study planning.

The total iron requirement recorded in patient charts had been calculated using the standard institutional formula:

$\text{Total iron (mg)} = 2.4 \times \text{weight (kg)} \times (\text{target hemoglobin} - \text{baseline hemoglobin}) + 5001$

The target hemoglobin was 11 g/dL. Hospital records showed that patients received 200-mg doses of iron sucrose (two 5 mL ampoules, diluted in 100 mL of normal saline) infused over 15-20 minutes on alternate days until the calculated requirement was met. All infusions were administered in an inpatient setting, and any documented infusion-related adverse events recorded by treating clinicians were included in the analysis. Hospital records indicated administration of the calculated total iron dose as per institutional protocol. No documented cases of premature discontinuation were identified. Incomplete dosing, if present, could not be separately analyzed due to reliance on archived documentation.

Data collection procedures

Data were extracted from antenatal case sheets, laboratory reports, infusion registers, and delivery records using a structured data abstraction format. Extracted variables included demographic characteristics (age, residence, and socioeconomic status), obstetric details (gravidity and gestational age), baseline hematologic parameters (hemoglobin, hematocrit, MCV, and serum proteins), and documented comorbidities. Laboratory investigations, such as complete blood counts, renal and liver function tests, urine and stool examinations, and thalassemia screening, were recorded as originally performed during routine clinical care.

Follow-up hematologic values at approximately six weeks post-treatment, along with delivery outcomes (mode of delivery and term/preterm status), neonatal outcomes (birth weight, gestational age at birth, and neonatal intensive care unit (NICU) admission), and any infusion-related adverse events were extracted exactly as documented in the archived records. No additional examinations or follow-ups were conducted for research purposes. Data abstraction was performed by trained investigators using a predefined structured format, with random cross-verification undertaken to minimize transcription errors.

Outcome measures

The primary outcome was the change in hemoglobin concentration between baseline and the six-week post-treatment value documented in the records. Secondary outcomes included changes in red-cell indices, maternal obstetric outcomes (mode and timing of delivery), neonatal outcomes (birth weight and NICU admission), and any infusion-related adverse events recorded in the case files. The six-week follow-up hematologic measurement was defined as a value recorded between five and seven weeks following completion of iron sucrose therapy. Maternal delivery outcomes and neonatal indicators were prespecified secondary outcomes.

Statistical analysis

Data were entered into a digital database and checked for completeness and accuracy using double-entry validation. Continuous variables were inspected for distributional characteristics, and normality was assessed using statistical and graphical methods. Descriptive statistics summarized participant characteristics, with continuous variables expressed as means with standard deviations or medians with interquartile ranges, and categorical variables expressed as frequencies and percentages. Normality of paired hematologic differences was assessed using the Shapiro-Wilk test prior to the application of paired t-tests. Because the study involved paired hematologic values documented at two time points in the same patients, comparisons between baseline and follow-up were performed using paired t-tests when assumptions were met. Categorical variables were compared using the chi-square test or Fisher’s exact test when appropriate. Effect sizes for the primary outcome were expressed as mean differences with 95% confidence intervals (CIs), and Cohen’s d was calculated to quantify the magnitude of change. All analyses used two-sided tests, and a p-value less than 0.05 was considered statistically significant. All infusions were administered under routine institutional monitoring protocols, with adverse events documented by treating clinicians in patient case records. Records with missing primary outcome data were excluded from analysis. Missing data in non-primary variables were handled using available-case analysis without imputation.

Ethical considerations

The study received approval from the Unique Hospital Multispeciality and Research Institute Ethics Committee in June 2020, with Protocol Number 20210626-SS, as a retrospective, record-based analysis. As all data were extracted from anonymized patient records and no direct contact with participants occurred, the requirement for informed consent was formally waived.

## Results

A total of 75 complete case records of pregnant women with IDA were identified and included in the analysis, with delivery and neonatal outcomes available in the archived files. The results are presented in a structured manner, with tables embedded at the points where corresponding findings are first described.

Baseline maternal characteristics

**Figure 1:**
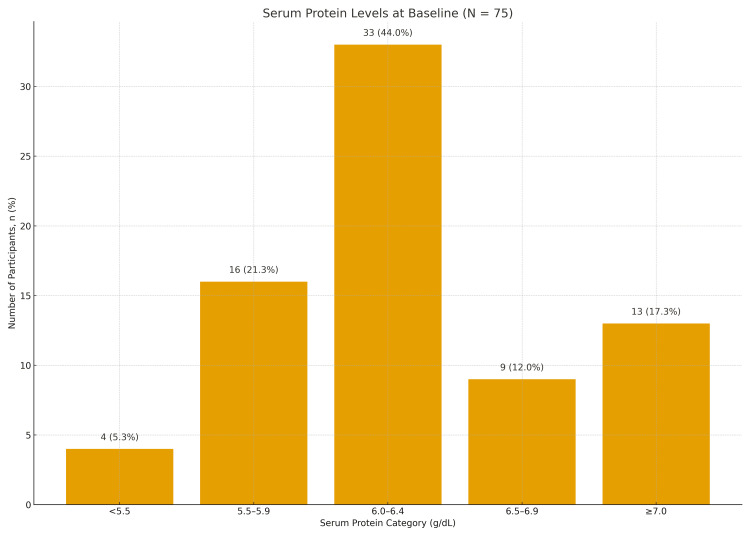
Serum Protein Levels at Baseline (N = 75) Bar chart showing the distribution of serum protein categories among study participants. Values are displayed as n (%). Statistical comparison performed using χ² test; significance defined as p < 0.05.

Baseline serum protein levels showed a predominance of hypoproteinemia, with the majority of women having values below 6.5 g/dL; the distribution across protein categories differed significantly (χ² = 22.34, p < 0.001). Mean baseline hemoglobin was 7.8 ± 0.6 g/dL, and mean baseline MCV was 67.8 ± 4.1 fL, consistent with moderate microcytic, hypochromic anemia. These findings are summarized in Table 1 and Figure 1.

**Table 1:** Baseline Nutritional and Hematologic Status Serum protein was analyzed using χ². Continuous variables (hemoglobin and MCV) are presented as mean ± SD. The significance threshold was p < 0.05.

Parameter	Category/Mean	N (%) or mean ± SD	Test statistic	p-value
Serum protein (g/dL)	<5.5	4 (5.3)	22.34	<0.001
	5.5-5.9	16 (21.3)	-	-
	6.0-6.4	33 (44.0)	-	-
	6.5-6.9	9 (12.0)	-	-
	≥7.0	13 (17.3)	-	-
Hemoglobin (g/dL)	Baseline	7.8 ± 0.6	-	-
Mean corpuscular volume (MCV) (fL)	Baseline	67.8 ± 4.1	-	-

Hematologic response to IV iron sucrose

**Figure 2:**
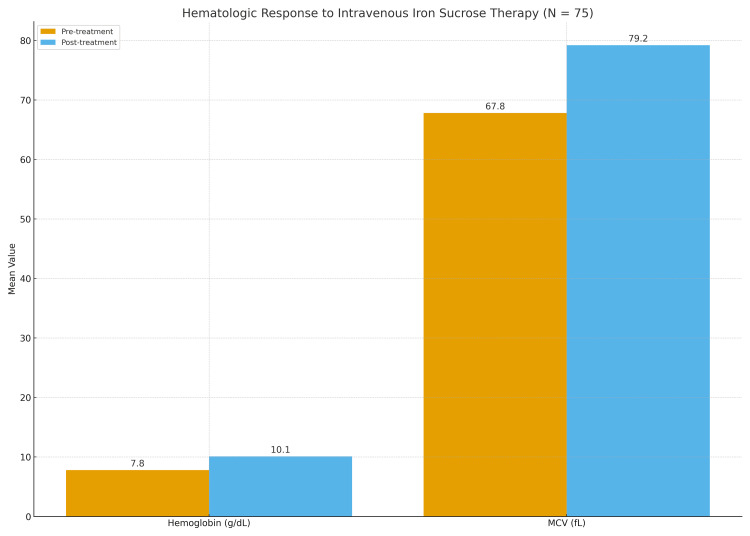
Hematologic Response to Intravenous Iron Sucrose Therapy (N = 75) Comparison of mean hemoglobin (g/dL) and mean corpuscular volume (MCV, fL) before and six weeks after intravenous iron sucrose therapy. Mean values are annotated above each bar. Statistical comparison was performed using a paired t-test, with significance defined as p < 0.05.

Six-week post-treatment hematologic values documented in the case records showed a marked and statistically significant improvement. Mean hemoglobin increased by 2.3 g/dL (95% CI: 2.2-2.4; p < 0.001), and mean MCV increased by 11.4 fL (p < 0.001), reflecting correction of iron-deficiency erythropoiesis. The magnitude of hemoglobin improvement demonstrated a large effect size (Cohen’s d = 1.9). These post-treatment responses are summarized in Table 2 and Figure 2.

**Table 2:** Hematologic Response to Intravenous Iron Sucrose Therapy Comparison of hemoglobin and MCV before and six weeks after intravenous iron sucrose therapy. A paired t-test was used for analysis. Statistical significance was set at p < 0.05. Mean change values are presented as mean difference ± standard deviation, with corresponding 95% confidence intervals for hemoglobin change reported in the Results section.

Parameter	Pre-treatment mean ± SD	Post-treatment mean ± SD	Mean change	t-value	p-value
Hemoglobin (g/dL)	7.8 ± 0.6	10.1 ± 0.7	+2.3 ± 0.4	14.92	<0.001
Mean corpuscular volume (MCV) (fL)	67.8 ± 4.1	79.2 ± 5.2	+11.4 ± 3.2	11.36	<0.001

Safety and tolerability

No serious adverse events were documented in the available medical records among the women included in the review. A few minor infusion-related reactions were documented in the case records, and one case of superficial thrombophlebitis was noted. No anaphylactic reactions were reported. The distribution of recorded events is shown in Table 3.

**Table 3:** Adverse Events Following Iron Sucrose Infusion Values are expressed as N (%). χ² was used for category comparison. The significance threshold was p < 0.05.

Adverse event	N (%)	χ²	p-value
Minor reactions	5 (6.7)	3.92	0.14
Thrombophlebitis	1 (1.3)	-	-
Anaphylaxis	0 (0.0)	-	-

Maternal delivery outcomes

Most women had full-term deliveries, and vaginal birth was the most frequently documented mode of delivery. Preterm delivery was recorded in a small proportion of cases. Delivery outcomes are shown in Table 4.

**Table 4:** Maternal Delivery Outcomes After Intravenous Iron Sucrose Therapy χ² was used for categorical analysis. Significance was defined as p < 0.05.

Outcome	N (%)	χ²	p-value
Full-term vaginal delivery	49 (65.3)	21.41	<0.001
Full-term cesarean section	19 (25.3)	-	-
Preterm delivery	7 (9.3)	-	-

Neonatal outcomes

Neonatal birth weights were generally favorable, with most infants weighing 2.5 kg or more. NICU admissions were infrequently documented. These outcomes are summarized in Table 5.

**Table 5:** Neonatal Birth Weight and Neonatal Intensive Care Unit (NICU) Admission Neonatal characteristics, including birth weight (kg) and NICU admission. χ² = chi-square test; statistical significance was defined as p < 0.05.

Parameter	Category	N (%)	χ²	p-value
Birth weight	<2.0 kg	7 (9.3)	17.26	0.004
	2.0-2.4 kg	8 (10.6)	-	-
	2.5-2.9 kg	42 (56.0)	-	-
	≥3.0 kg	18 (24.0)	-	-
NICU admission	Yes	5 (6.7)	-	-
	No	70 (93.3)	-	-

## Discussion

This retrospective, observational study evaluated the hematologic response, maternal outcomes, and neonatal parameters following intravenous iron sucrose therapy in pregnant women with moderate to moderately severe IDA in late gestation.

The findings demonstrate substantial improvements in hemoglobin and red-cell indices, a low rate of adverse reactions, and reassuring perinatal outcomes. These results add context-specific evidence to the growing body of literature supporting intravenous iron therapy in populations with a high anemia burden and limited time for correction.

The baseline demographic and nutritional profile of the cohort - characterized by younger maternal age, lower socioeconomic background, microcytic, hypochromic anemia, and widespread hypoproteinemia - mirrors longstanding epidemiologic patterns reported in the region and globally [11-13]. These features highlight the chronic nutritional and physiological stressors that contribute to IDA in pregnancy. The presence of hypoproteinemia further supports an underlying nutrition-related deficiency, consistent with mechanisms described in earlier literature [14,15].

Based on documented treatment records, hemoglobin increased significantly, accompanied by a notable rise in MCV. This magnitude of hematologic improvement aligns with findings from recent Indian and international studies, demonstrating rapid and clinically meaningful correction with intravenous iron compared with oral formulations [12,16]. The rapidity of response is especially relevant in late-gestation anemia, where treatment windows are narrow, and oral iron absorption is often insufficient to achieve timely recovery [11,14].

The safety profile observed in this study is consistent with extensive evaluations of iron sucrose, which report minimal infusion-related adverse events and a very low risk of anaphylaxis. Prior work has repeatedly confirmed the high tolerability of iron sucrose, attributed to its non-dextran composition and predictable immunological behavior [16-18]. The absence of severe hypersensitivity reactions and the rarity of infusion complications in the present cohort reinforce its suitability for routine antenatal use.

Maternal and neonatal outcomes documented in the records were favorable, with most women achieving term delivery and the majority of newborns weighing ≥2.5 kg. These results align with evidence indicating that effective maternal anemia correction supports placental function, fetal oxygenation, and improved neonatal growth trajectories [12-14]. Although causality cannot be inferred due to the observational design, the consistency of these outcomes with prior studies strengthens the hypothesis that timely anemia correction contributes to improved perinatal stability.

The hematologic outcomes observed in this study parallel previous reports documenting hemoglobin increases of 2-3 g/dL with intravenous iron sucrose among moderately anemic pregnant women [12,13,16]. Comparative analyses have consistently shown that intravenous iron provides faster and more reliable correction than oral therapy, particularly in women presenting during the second or third trimester [14]. The safety findings similarly reflect those of clinical trials, demonstrating low rates of serious adverse events and distinguishing iron sucrose from older dextran-based preparations [15,19].

The maternal and neonatal outcomes reported here - high rates of term delivery, adequate birth weight distribution, and low NICU admission - are consistent with evidence from population-based studies and meta-analyses supporting the broader benefits of maternal iron optimization [12,13,18]. Favorable maternal and neonatal outcomes were observed; however, given the single-arm, retrospective design, these findings represent associations rather than causal treatment effects.

Theoretical and practical implications

The results reinforce the biological rationale for intravenous iron sucrose in pregnancy. By delivering readily bioavailable iron directly into the reticuloendothelial system, iron sucrose enables rapid erythropoietic recovery, bypassing gastrointestinal limitations that commonly hinder oral iron therapy. Practically, this makes IV iron particularly valuable in late-gestation anemia, in women with intolerance to oral iron, and in regions with high anemia prevalence.

In resource-limited settings, where nutritional deficiencies and late antenatal visits are common, the ability of intravenous iron to achieve timely hematologic correction may reduce the burden of transfusion, lower preterm birth risk, and improve neonatal outcomes. The findings, therefore, have direct implications for strengthening antenatal anemia management protocols in similar populations.

Study limitations

This study has several limitations. The single-arm, non-randomized design prevents direct comparison with oral iron or alternative intravenous formulations, limiting causal interpretation. Iron biomarkers such as ferritin, transferrin saturation, or reticulocyte hemoglobin were not assessed, restricting mechanistic insight into iron store repletion. The sample size, while adequate for detecting hematologic changes, may not capture rare maternal or neonatal adverse events. Additionally, the single-center setting may limit generalizability to populations with different nutritional or socioeconomic characteristics. As this was a retrospective, record-based analysis, data completeness depended on the accuracy and availability of archived records, and unmeasured confounding cannot be excluded. While iron deficiency was inferred from hemoglobin concentration and microcytic red-cell indices, the absence of iron studies such as serum ferritin means that other causes of microcytic anemia cannot be completely excluded. Iron deficiency could not be biochemically confirmed due to the absence of serum ferritin or iron studies in archived records. Consequently, hemoglobinopathies such as thalassemia trait could not be definitively excluded. Inclusion of such cases may have attenuated the observed hematologic response, as these conditions do not respond to iron therapy.

As adverse events were identified through routine clinical documentation, minor infusion-related reactions may have been underreported compared with prospective active surveillance.

Despite these limitations, the study offers robust, real-world clinical insights and provides valuable data from a nutritionally vulnerable population, where evidence on parenteral iron therapy remains limited.

Future research directions

Future studies should include larger, randomized controlled trials comparing iron sucrose with newer intravenous formulations, such as ferric carboxymaltose. Longitudinal assessments of neonatal and childhood outcomes would help clarify the long-term benefits of maternal iron correction. Incorporating biochemical markers and placental perfusion assessments may also elucidate mechanistic pathways linking maternal iron status to fetal development. Additionally, evaluating patient-reported outcomes - including functional capacity and quality of life - may further contextualize the clinical impact of anemia correction during pregnancy.

## Conclusions

This retrospective, record-based analysis demonstrates that intravenous iron sucrose is associated with a clinically meaningful rise in hemoglobin levels among pregnant women with moderate microcytic anemia in late gestation. No serious adverse events were documented in the available records, indicating a favorable tolerability profile under routine clinical use. Maternal and neonatal outcomes observed in this cohort were reassuring, but should be interpreted as associations rather than causal effects due to the study design.
